# Harvesting PdH Employing Pd Nano Icosahedrons via High Pressure

**DOI:** 10.1002/advs.202205133

**Published:** 2022-11-14

**Authors:** Kun Shi, Zihao Huo, Tianxiao Liang, Yongming Sui, Chuang Liu, Haiyun Shu, Lin Wang, Defang Duan, Bo Zou

**Affiliations:** ^1^ State Key Laboratory of Superhard Materials College of Physics Jilin University Changchun 130012 P. R. China; ^2^ Synergetic Extreme Condition User Facility State Key Laboratory of Superhard Materials College of Physics Jilin University Changchun 130012 P. R. China; ^3^ Center for High Pressure Science and Technology Advanced Research Shanghai 211203 P. R. China; ^4^ Center for High Pressure Science (CHiPS) State Key Laboratory of Metastable Materials Science and Technology Yanshan University Qinhuangdao Hebei 066004 P. R. China

**Keywords:** palladium hydrides, quench, high pressure, preferential orientation of facets, potential barrier

## Abstract

Palladium hydrides (PdH*_x_*) have important applications in hydrogen storage, catalysis, and superconductivity. Because of the unique electron subshell structure of Pd, quenching PdH*_x_* materials with more than 0.706 hydrogen stoichiometry remains challenging. Here, the 1:1 stoichiometric PdH ($Fm\bar{3}m)$ is successfully synthesized using Pd nano icosahedrons as a starting material via high‐pressure cold‐forging at 0.2 GPa. The synthetic initial pressure is reduced by at least one order of magnitude relative to the bulk Pd precursors. Furthermore, PdH is quenched at ambient conditions after being laser heated ≈2000 K under ≈30 GPa. Corresponding ab initio calculations demonstrate that the high potential barrier of the facets (111) restricts hydrogen atoms' diffusion, preventing hydrogen atoms from combining to generate H_2_. This study paves the way for the high‐pressure synthesis of metal hydrides with promising potential applications.

## Graphical abstract

Combining the laser heating‐assisted high‐pressure synthesis and the morphology modification of Pd nanomaterials, successfully synthesize and quench PdH in $Fm\bar{3}m$. Corresponding ab initio calculations demonstrate that the potential barriers of different facets affect the hydrogen diffusion to different degrees. This work opening the door for potential industrial application in hydrogen storage of Pd.

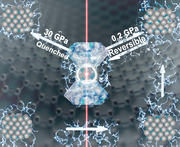

## 1 Introduction

Palladium hydride (PdH*_x_*) has been enthusiastically studied for more than 150 years, because the Pd−H system plays a critical role in many areas such as catalysis^[^ ^1^ ^]^ and superconductivity.^[^ ^2^ ^]^ Palladium can absorb nearly a thousand times its own volume of hydrogen under easily reachable pressure and temperature.^[^ ^3^ ^]^ However, limited by experimental technology, the increase in hydrogen content of more than 0.706 has entered a long bottleneck period. High‐pressure synthesis, as a clean, effective, and versatile technique, can provide a way to attain the H‐rich metal hydrides. Research on PdH*_x_* has made some limited progress through the introduction of high‐pressure synthesis technology. The 1:1 stoichiometric PdH was synthesized at 3.8 GPa using bulk palladium as the starting material. Although the sample was pressurized up to 100 GPa and laser heated several times, the PdH failed to be quenched after recovering the pressure and temperature to the ambient conditions.^[^ ^4^ ^]^

Compared with bulk materials, nanomaterials show higher chemical activity due to quantum size and surface effects.^[^ ^5^ ^]^ Taking iridium (Ir) as an example, in the range of 0 to 100 ppm hydrogen pressure, the hydrogen absorption of Ir nanoparticles increased with increasing hydrogen pressure, while the bulk Ir did not exhibit any hydrogen absorption.^[^ ^6^ ^]^ Several recent studies have shown that, in addition to size, shape is also an important factor affecting the properties of nanomaterials.^[^ ^7^ ^]^ For instance, using rhodium (Rh) nano icosahedrons and Rh nano cubes of similar size as precursors, the synthetic initial pressures of RhH were 3.5 and 4.4 GPa, respectively. This is because nano icosahedrons consist of 20 (111) tetrahedral units and 30 twin boundaries, which reduce the enthalpy of formation.^[^ ^8^ ^]^ Inspired by the above, using nanosized palladium with characteristic facets as precursors is expected to design metal hydrides with more hydrogen content.

Herein, we synthesized PdH ($Fm\bar{3}m)$ at a very low pressure (≈200 MPa) using Pd nano icosahedrons as a starting material via high‐pressure cold‐forging. For 1:1 stoichiometric PdH, it absorbed ≈1300 times its own volume of H_2_, opening the door for potential industrial application in hydrogen storage. Upon further increasing pressure to ≈30 GPa with laser heating to ≈2000 K, PdH is quenched after recovering the pressure and temperature to ambient conditions. This achievement increases the hydrogen content of PdH*_x_* that can be retained at ambient conditions by ≈40%. Intriguingly, PdH exhibits preferential orientation of facets (111). First‐principles calculations indicate that the (111) facet has a high potential barrier, which can effectively restrict the diffusion of hydrogen atoms, thus making a great contribution to the quenchable PdH. This study offers a robust strategy to harvest H‐rich transition metal hydrides with potential practical applications.

## 2 Results and Discussion

**Figure 1:**
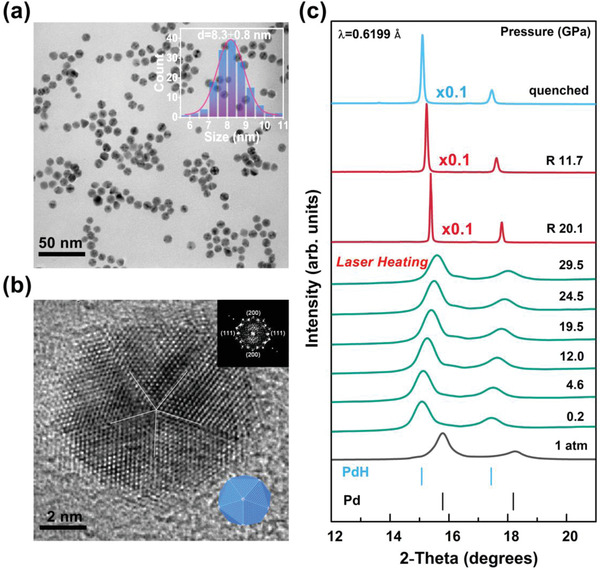
a) Scanning transmission electron microscopy (STEM) image of the Pd nano icosahedrons. The inset image is the SAED of the representative nano icosahedrons in this area. b) High‐resolution transmission electron microscopy (HRTEM) image of an individual icosahedron. The inset image is the corresponding fast Fourier transform (FFT) pattern and geometric model. c) Representative in situ ADXRD patterns at selected pressures of the Pd nano icosahedrons and hydrogen system in the presence of H_2_ as PTM. Pure phase of Pd nano icosahedrons (black line), unheated phase (green line), phase after heating (red line), and pure phase of PdH (blue line).

Pd nano icosahedrons (**Figure** **1**a) were prepared via the wet‐chemical reduction method (see Supporting Information for details). Scanning transmission electron microscopy (STEM) image shows that highly uniform Pd nano icosahedrons with an average size of 8.3 ± 0.8 nm (inset of Figure 1a) were obtained. As shown in Figure 1b, by measuring the distance between the diffraction spots in the fast Fourier transform (FFT) image of high‐resolution transmission electron microscopy (HRTEM), the average interplanar spacing of the facets (111) and (200) is calculated to be 0.226 and 0.194 nm, respectively. At ambient conditions, the peaks centered at 2*θ* of ≈15.78° and ≈18.21° (bottom of Figure 1c) belong to the (111) and (200) facets of pure Pd nano icosahedrons with face‐centered cubic (fcc) structure, respectively. After hydrogen was loaded at the initial pressure of 0.2 GPa, the diffraction peaks move to a lower diffraction angle, indicating hydrogen atoms have entered the lattice and the cubic phase PdH (space group: $Fm\bar{3}m$, a = 4.10 Å) with step of 14.7% unit‐cell volume expansion (Δ*V* = 2.46 Å^3^) is formed. This finding is an order of magnitude lower than the synthetic initial pressure of 3.8 GPa for the bulk Pd as precursors.^[^ ^4^ ^]^ Upon further increasing pressure to 29.5 GPa, the diffraction peak gradually moves to a higher angle due to the compression of the crystal lattice. After laser heating to about 2000 K, the diffraction peak intensity suddenly increased and became sharp. As the pressure decreased, the relative intensity of the diffraction peak significantly changed. After decompression, PdH is quenched.

**Figure 2:**
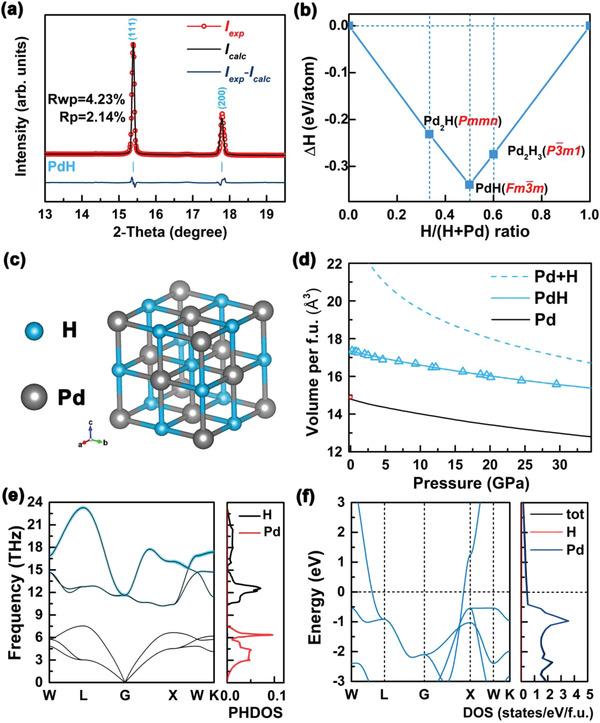
a) Structural refinement of the ADXRD pattern recorded at a pressure of 20.1 GPa after laser heating at ≈2000 K. The experimental data, full‐profile refinement, and difference pattern are shown as red, black, and black–blue curves, respectively. The tick marks for PdH indicate the theoretical peak positions calculated from the refined lattice parameters. b) Formation enthalpies of predicted PdH*_x_* compounds with respect to their decomposition into Pd and H at 20 GPa. The *Fm* $\bar{3}$ *m* structure for Pd and the *P63/mc* structures for hydrogen were adopted. c) Illustrations for the crystal structures of PdH. d) Equations of states (EOS) of Pd,^[^ ^4^ ^]^ PdH, and Pd + H are presented by black, blue line, and dash line, respectively. Experimental data for Pd and PdH are shown by red circle and blue triangle, respectively. e) Calculated phonon dispersion curves and phonon density of states (DOS) for PdH at 20 GPa. f) Electronic band structure and projected DOS for PdH at 20 GPa.

Rietveld refinement of the ADXRD pattern was performed to prove the correctness of the palladium‐hydride structure. As presented in **Figure** **2**a, the refinement profile matches well with the experimental data at 20.1 GPa (Rwp = 4.23%, *R*p = 2.14%). The ab initio calculations were carried out to understand the detailed nature of the Pd−H system at high pressure, including thermodynamic stability, dynamic stability, and electronic structures. PdH is located on the convex hull, which implies it is a thermodynamically stable phase (Figure 2b). Pd atoms occupy the fcc sublattice in the structure of PdH, whereas H atoms fill the octahedral positions (Figure 2c). Detailed predicted structural parameters are shown in Table S1 (Supporting Information). Most transition‐metal hydrides exhibit a closed‐packed metal host lattice with hydrogen atoms occupying the octahedral or tetrahedral interstitial sites. The synthetic initial pressures of these metal hydrides are generally high, e.g., 15 GPa for RuH^[^ ^9^ ^]^ and 27 GPa for PtH.^[^ ^10^ ^]^ In our experiments, PdH was synthesized under very low pressure, revealing that Pd nano icosahedrons had better hydrogen‐binding characteristics.^[^ ^8b^ ^]^

Furthermore, the pressure–volume (*P*–*V*) curves were fitted by third‐order Birch–Murnaghan equation:^[^ ^11^ ^]^

(1) $$\begin{array}{ccc} P & = & \frac{3B_{0}}{2}\left[{\left(\frac{V}{V_{0}}\right)}^{-\frac{7}{3}}-{\left(\frac{V}{V_{0}}\right)}^{-\frac{5}{3}}\right]\\ & & \times \left\{1+\frac{3}{4}\left(B_{0}^{{}^{\prime }}-4\right)\left[{\left(\frac{V}{V_{0}}\right)}^{-\frac{2}{3}}-1\right]\right\} \end{array}$$

where *V* is the volume per formula unit at given pressure *P*, *V*_0_ is the volume per formula unit at ambient pressure, *B*_0_ is the bulk modulus, and $B_{0}^{\prime }$ is the first pressure derivative of *B*_0_. The fitted *P*–*V* curves of PdH*_x_* are plotted in Figure 2d together with experimental data points. At 20.1 GPa, the experimental volume of PdH is 15.95 Å^3^ f.u.^−1^, which is in good agreement with the theoretical values. As shown in Figure 2e, no imaginary phonon frequencies were found in the entire Brillouin zone, demonstrating the dynamic stability of this structure. The electronic band structure and projected density of states (DOS) as shown in Figure 2f clearly reveal that PdH presents metallicity because of the conduction and valence bands overlap at the Fermi level.

**Figure 3:**
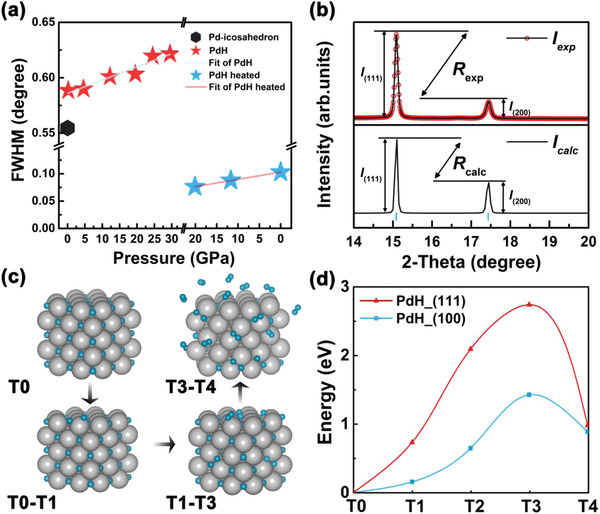
a) Pressure−full width at half maximum (FWHM) diagram of Pd and PdH at selected pressures. b) The experimental and the calculated data of the diffraction peak intensity ratio corresponding to the (111) and (200) facets at ambient conditions. c) Schematic representation of hydrogen escape from Pd−H system during depressurization. d) Computational simulation of the potential barriers to cross for hydrogen desorption from different facets at ambient condition.

Analyzing the hydrogen diffusion path in the Pd lattice can provide a theoretical foundation for improving the hydrogen storage performance of PdH*_x_*. The full width at half maximum (FWHM) of the diffraction peaks rises slightly during the PdH generation process, as shown in **Figure** **3**a. It means that high‐pressure cold‐forging did not disrupt the original structure of Pd nano icosahedrons. After laser heating, the FWHM of the peaks dropped by an order of magnitude. It can be assumed that the PdH sintered to form larger‐sized crystals.^[^ ^12^ ^]^ Furthermore, there is a significant divergence between the experimental and calculated data of the diffraction peak intensity ratio corresponding to the (111) and (200) facets ( $R_{\mathrm{calc}}=\frac{I_{\left(111\right)}}{I_{\left(200\right)}}=$1.9; $R_{\exp}=\frac{I_{\left(111\right)}}{I_{\left(200\right)}}=$5.3). This result shows that the PdH produced experimentally has a preferential orientation of the (111) facets. The structural refinement of PdH (Figure S1, Supporting Information) indicates that the lattice is nonstandard, which might be produced by residual stress in the lattice during the cooling process at high pressure.

It is generally accepted that hydrogen desorption is a process in which a hydrogen atom diffuses to an adjacent position and recombines with another hydrogen atom to form H_2_, which then escapes from the former system. Previous research has shown that the facets (111) are more conducive to hydrogen adsorption than other facets.^[^ ^7a^ ^]^ Accordingly, we compared the diffusional barriers of different facets, namely (111) and (100), by simulating and calculating the above‐mentioned hydrogen motion behavior (Figure 3c), to explain the mechanism of PdH retention. The initial state (T_0_) is that each atom of PdH exists stably in the equilibrium position, and the final state (T_4_) means the adjacent hydrogen atoms have been recombined into H_2_ molecules. The transition states (T_1_ − T_3_) were obtained by the climbing‐image nudged elastic band (CI‐NEB) method. These findings in Figure 3d clearly reveal that the barrier energy required for hydrogen atoms to desorb from the (111) facets is substantially larger than that required for the (100) facets. Therefore, we assume that during the depressurization process, due to the presence of a large number of (111) facets in the system, a high potential barrier to restrict hydrogen atom diffusion is generated, resulting in hydrogen atoms being unable to combine to form H_2_ and escape the system.

## 3 Conclusion

In summary, we report the successful synthesis of the 1:1 stoichiometric PdH with $Fm\bar{3}m$ symmetry at a mild pressure of 0.2 GPa by employing Pd nano icosahedrons as a starting material. After increasing the pressure to 30 GPa and laser heating, PdH exhibits preferential orientation of facets (111). Through computational simulation of the hydrogen desorption process, we give evidence that the (111) facet has a high potential barrier, which can effectively restrict the diffusion of hydrogen atoms, thus achieving the retention of PdH. Our work elucidates the pathways and mechanisms of hydrogen diffusion and desorption in Pd−H systems, providing a reference for future design and research of hydrogen storage materials.

## Conflict of Interest

The authors declare no conflict of interest.

## Supporting information

Supporting Information

## Data Availability

Research data are not shared.
