# Supplementary material for: Harvesting PdH Employing Pd Nano Icosahedrons via High Pressure
Source: Adv Sci (Weinh). 2022 Nov 14;10(4):2205133. doi: 10.1002/advs.202205133 (PMC9896048; doi:10.1002/advs.202205133)
Supplement: Supplementary file 1 — Supporting Information [file ADVS-10-2205133-s001.pdf]

ADVANCED SCIENCE
Open Access

Supporting Information

for *Adv. Sci.*, DOI 10.1002/advs.202205133

Harvesting PdH Employing Pd Nano Icosahedrons via High Pressure

Kun Shi, Zihao Huo, Tianxiao Liang, Yongming Sui, Chuang Liu, Haiyun Shu, Lin Wang, Defang Duan* and Bo Zou**

Supporting Information

Harvesting PdH Employing Pd Nano Icosahedrons via High Pressure

Kun Shi, Zihao Huo, Tianxiao Liang, Yongming Sui, Chuang Liu, Haiyun Shu, Lin Wang, Defang Duan,* and Bo Zou**

K. Shi, Z. Huo, T. Liang, Prof. Y. Sui, Prof. D. Duan, Prof. B. Zou
State Key Laboratory of Superhard Materials, College of Physics, Jilin University, Changchun 130012, China
C. Liu
Synergetic Extreme Condition User Facility, State Key Laboratory of Superhard Materials, College of Physics, Jilin University, Changchun 130012, China
H. Shu
Center for High Pressure Science and Technology Advanced Research, Shanghai 211203, China
L. Wang
Center for High Pressure Science (CHiPS), State Key Laboratory of Metastable Materials Science and Technology, Yanshan University, Qinhuangdao, Hebei 066004, China
Y. Sui
State Key Laboratory of Superhard Materials, College of Physics, Jilin University, Changchun 130012, China.
Email: suiym@jlu.edu.cn
D. Duan
State Key Laboratory of Superhard Materials, College of Physics, Jilin University, Changchun 130012, China.
Email: duandf@jlu.edu.cn
B. Zou
State Key Laboratory of Superhard Materials, College of Physics, Jilin University, Changchun 130012, China.
Email: zoubo@jlu.edu.cn

Method

Diamond anvil cell (DAC) with flat anvil surface of about 300 μm in diameter as a pressure vehicle was adjusted to parallel alignment. Rhenium gasket was pre-indented about 35 μm thickness, and a hole 200 μm in diameter was cutted by laser drilling. Ruby luminescence pressure scale for quasi-hydrostatic condition was mounted on the anvil surface. Excess liquid hydrogen was loaded into the gasket hole at room temperature as reagent and pressure transmitting medium. After the initial clamping of hydrogen in a DAC, the compression force was increased in steps by a lever mechanism. *In situ* angle-dispersive X-ray diffraction (ADXRD) patterns were recorded on the BL15U1 synchrotron beamline at Shanghai Synchrotron Research Facility. The sample in the DAC cavity was heated by the double-sided laser heating system (IPG model YLR-100-AC-Y11) at Center for High Pressure Science & Technology Advanced Research, when the pressure reaches about 30 GPa.

Calculational details

The *ab initio* random structure searching methods were used to predict the stable or metastable structures of Pd-H system, as implemented in the AIRSS (Ab Initio Random Structure Searching) code[1] combining with CASTEP (Cambridge serial total energy package) code[2]. The equations of states and electronic properties were performed by the VASP (Vienna ab initio simulation packages) code[3], where the Perdew–Burke–Ernzerhof [4] of generalized gradient approximation[5] with the all-electron projector-augmented wave method[6] was chosen. The electron-ion interaction was described with the $1s^1$ and $4s^24p^64d^{10}$ configurations treated as valence electrons for H and Pd, respectively. The cutoff energy of 800 eV and k-point spacing of $2\pi\times0.03$ Å^{-1} were adopted to ensure the enthalpy converges to less than 1 meV/atom. The climbing-image nudged elastic band (CI-NEB) method[7] was employed to find the minimum-energy paths of hydrogen desorption at different facets. The phonon calculations of PdH were carried out within the framework of the linear-response theory via the QUANTUM ESPRESSO package[8]. Convergence tests show that 70 Ry is a suitable cutoff energy for the plane-wave basis set. The *q*-point and *k*-point mesh in the first Brillouin zone of 5×5×5 and 20×20×20 were used.

Synthesis of Pd nano icosahedrons

In a procedure for the synthesis of Pd nano icosahedrons, 17.73 mg of $PdCl_2$, 100 mg of PVP and 10ml of EG were added into a 15 ml Erlenmeyer flask. Use an ultrasonic cleaner and a magnetic stirrer to mix the reactants in the Erlenmeyer flask thoroughly, then add 165 μL of concentrated HCl. The mixed solution was heated in an oil bath at 165 °C under magnetic stirring. Several minutes later, the solution gradually becomes clear and 90 μL of concentrated

HCl was injected. After about 5 minutes, take out the Erlenmeyer flask and rinse it with cold water. Finally, the Pd nano icosahedrons were collected by high speeding centrifugation (15000 rpm) after washed once with acetone and thrice with DI water to remove excessive PVP. The chemicals used in the synthesis were purchased from Alfa Aesar.

Table S1 | Structural parameters of Pd-H compounds

<table>
<tr><th rowspan="2">Compounds</th><th rowspan="2">Pressure
(GPa)</th><th rowspan="2">Space Groups</th><th rowspan="2">Lattice
Parameters</th><th colspan="2">Fractional Atomic Coordinates</th></tr>
<tr><th>Sites</th><th>Coordinates</th></tr>
<tr><td rowspan="2">PdH</td><td>0</td><td>$Fm\overline{3}m$</td><td>a=b=c=4.1125 Å
α=β=γ=90°</td><td>Pd (4a)
H (4b)</td><td>(0, 0, 0)
(0.5, 0, 0)</td></tr>
<tr><td rowspan="3">20</td><td>$Fm\overline{3}m$</td><td>a=b=c=4.0037 Å
α=β=γ=90°</td><td>Pd (4a)
H (4b)</td><td>(0, 0, 0)
(0.5, 0, 0)</td></tr>
<tr><td>Pd_2H</td><td>$Pmmn$</td><td>a=2.7572 Å
b=3.9358 Å
c=5.4934 Å
α=β=γ=90°</td><td>Pd (2b)
Pd (2b)
H (2a)</td><td>(0, 0.5, 0.38)
(0, 0.5, 0.871)
(0.5, 0.5, 0.136)</td></tr>
<tr><td>Pd_2H_3</td><td>$P\overline{3}m1$</td><td>a=b=2.9331 Å
c=4.8632 Å
α=β=90°
γ=120°</td><td>Pd (2d)
H (2d)
H (1a)</td><td>(0.667, 0.333, 0.241)
(0.333, 0.667, 0.62)
(0.0, 0.0, 0.0)</td></tr>
</table>

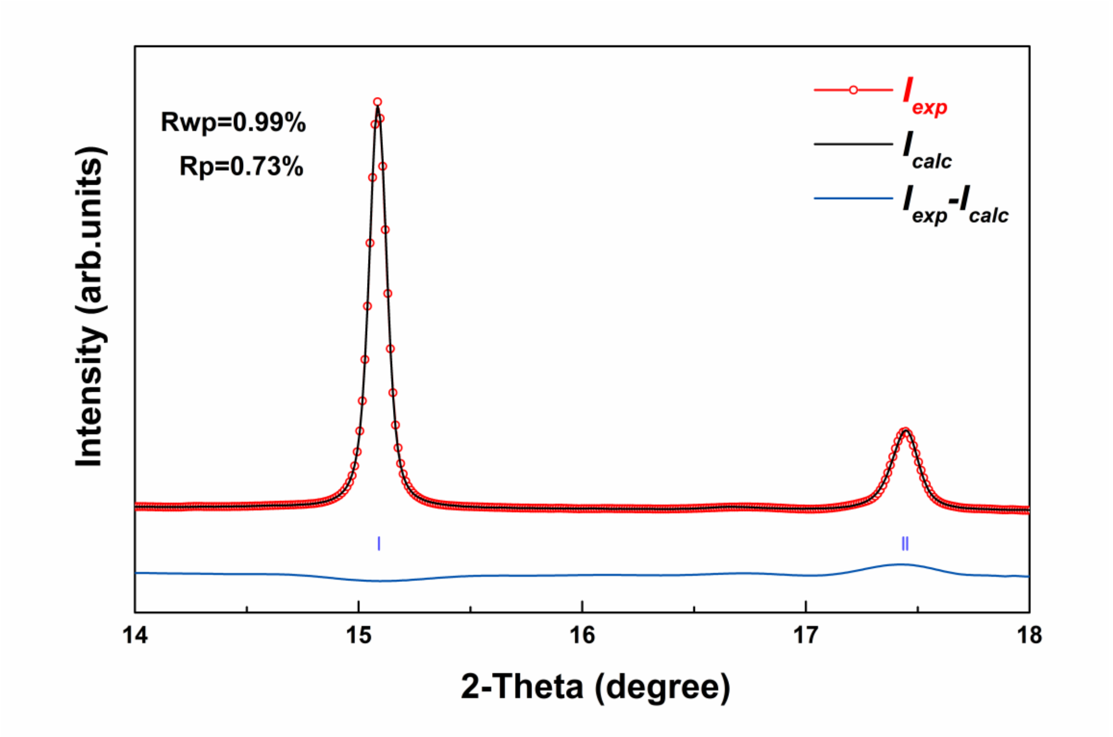

Figure S1. Structural refinement of the PdH ADXRD pattern recorded at ambient conditions.

The structural refinement indicates that PdH (space group: $Fm\overline{3}m$, a = 4.11 Å) with step of 17.3% unit-cell volume expansion (ΔV= 2.56 Å^3) is quenched at ambient conditions.

[1] a)C. J. Pickard, R. J. Needs, *Phys. Rev. Lett.* **2006**, 97, 045504; b)C. J. Pickard, R. J. Needs, *J. Phys.: Condens. Matter* **2011**, 23, 053201.
[2] M. D. Segall, P. J. D. Lindan, M. J. Probert, C. J. Pickard, P. J. Hasnip, S. J. Clark, M. C. Payne, *J. Phys.: Condens. Matter* **2002**, 14, 2717.
[3] G. Kresse, J. Furthmüller, *Phys. Rev. B* **1996**, 54, 11169.
[4] J. P. Perdew, K. Burke, M. Ernzerhof, *Phys. Rev. Lett.* **1996**, 77, 3865.
[5] J. P. Perdew, Y. Wang, *Phys. Rev. B Condens. Matter* **1992**, 46, 12947.
[6] P. E. Blochl, *Phys. Rev. B Condens. Matter* **1994**, 50, 17953.
[7] a)G. Henkelman, B. P. Uberuaga, H. Jónsson, *J. Chem. Phys.* **2000**, 113, 9901; b)G. Henkelman, H. Jónsson, *J. Chem. Phys.* **2000**, 113, 9978.
[8] P. Giannozzi, S. Baroni, N. Bonini, M. Calandra, R. Car, C. Cavazzoni, D. Ceresoli, G. L. Chiarotti, M. Cococcioni, I. Dabo, A. Dal Corso, S. de Gironcoli, S. Fabris, G. Fratesi, R. Gebauer, U. Gerstmann, C. Gougoussis, A. Kokalj, M. Lazzeri, L. Martin-Samos, N. Marzari, F. Mauri, R. Mazzarello, S. Paolini, A. Pasquarello, L. Paulatto, C. Sbraccia, S. Scandolo, G. Sclauzero, A. P. Seitsonen, A. Smogunov, P. Umari, R. M. Wentzcovitch, *J. Phys.: Condens. Matter* **2009**, 21, 395502.
